# Impact of Off-Time on Quality of Life in Parkinson's Patients and Their Caregivers: Insights from Social Media

**DOI:** 10.1155/2022/1800567

**Published:** 2022-12-03

**Authors:** Philippe Damier, Emily J. Henderson, Jesús Romero-Imbroda, Laura Galimam, Nick Kronfeld, Tobias Warnecke

**Affiliations:** ^1^CHU Nantes, INSERM, Nantes Université, CIC 1314, Nantes, France; ^2^Royal United Hospitals Bath NHS Foundation Trust, Combe Park, Bath, UK; ^3^Department of Neurology, Hospital Quirónsalud Málaga, Málaga, Spain; ^4^Lumanity, London, UK; ^5^Kyowa Kirin Co., Ltd., London, UK; ^6^Department of Neurology and Neurorehabilitation Klinikum, Osnabrück, Academic Teaching Hospital of the Westfälische Wilhelms-University of Münster, Osnabrück, Germany

## Abstract

**Introduction:**

In Parkinson's disease (PD), the quality of life of both patients and caregivers is affected. While key issues relating to quality of life may not emerge in conversations with healthcare professionals (HCPs), unguarded social media conversations can provide insight into how people with Parkinson's disease (PwPD) and their caregivers are affected. We conducted a qualitative and quantitative netnographic study of PD conversations posted on social media sites over a 12-month period.

**Objective:**

To identify key themes and issues for PwPD.

**Methods:**

Using predefined and piloted search terms, we identified 392,962 social media posts (between March 31, 2020, and March 31, 2021, for the UK and France, and between September 30, 2019, and March 31, 2021, for Italy, Spain, and Germany). A random sample of these posts was then analyzed using natural language processing (NLP), and quantitative, qualitative,in-depth contextual analysis was also performed.

**Results:**

Key themes that emerged in the PD conversation related to the changing experience of symptoms over time are the physical, emotional, and cognitive impact of symptoms, the management and treatment of PD, disease awareness among the general public, and the caregiver burden. The emotional impact of motor symptoms on PwPD is significant, particularly when symptoms increase and PwPD lose their independence, which may exacerbate existing anxiety and depression. Nonmotor symptoms can also compound the difficulties with managing the physical impact of motor symptoms. The burden of nonmotor symptoms is felt by both PwPD and their caregivers, with the impact of nonmotor symptoms on cognitive processes particularly frustrating for caregivers. The experience of off-time was also featured in the online conversation. Some PwPD believe there is a lack of adequate management from healthcare professionals, who may not appreciate their concerns or take sufficient time to discuss their needs.

**Conclusion:**

This study identified key themes that PwPD and their caregivers discuss online. These findings help signpost issues of importance to PwPD and areas in which their care may be improved.

## 1. Introduction

Parkinson's disease (PD) is a neurodegenerative disorder characterized by progressive motor and nonmotor symptoms. [1, 2] Globally, in 2016, approximately 6.1 million individuals had PD, and 211,296 deaths were attributed to PD. [3] Both motor symptoms, such as bradykinesia, rigidity, postural instability, and resting tremor, [3] and nonmotor symptoms, such as cognitive impairment, [2] sleep disturbance, [4] pain, [5] and depression, [6] have well-documented adverse effects on patient quality of life throughout the course of the illness. [7–10] The burden of PD is shared by caregivers, with this role recognized as associated with impaired psychosocial functioning and increasing psychiatric morbidity [11].

Although response to dopamine-replacement therapy (levodopa and/or dopamine agonist) is initially good, it often fluctuates as PD progresses [12]. Following the initial control of symptoms, over time, patients may experience periods when symptoms return between medication doses despite optimization of the dopaminergic regimen [13]. The return of symptoms is thought to occur due to progressive loss of dopamine neurons that results in functional impairment of dopamine storage [13] and to complex changes in the functioning of basal ganglia neurons [14]. “Off-time”—as these periods of symptomatic fluctuations are known—typically present as a reemergence of both motor and nonmotor symptoms [13, 14].

The clinical burden of off-time is significant; the number of people with Parkinson's disease (PwPD) who experience off-time increases from less than 5% after 7–12 months of treatment to approximately 40% after 4–6 years, and approximately 70% after 9 years or more [15]. In addition to impacting quality of life [16], off-time may present a clinical challenge to both patients and healthcare professionals (HCPs) [17]. Some side effects of dopaminergic treatment, such as levodopa-induced dyskinesia (in which the dopamine level fluctuations in combination with the loss of dopaminergic neurons can result in abnormal and involuntary movements) and neuropsychiatric manifestations (such as decreased reaction time, impulse control disorders, and excessive sleep) [18, 19] are, for patients and caregivers, sometimes difficult to differentiate from off-time, which might present similar symptoms.

Social media is a powerful communication tool that enables people to connect around topics of common interest [20]. With regard to PD, social media provide a mechanism for both PwPD and their caregivers to share their experiences and interact with other PwPD, caregivers, and a wider network of support that includes patient charities and advocacy groups. In this capacity, social media represents a unique portal into unguarded, personal healthcare experiences and an important resource to further understanding of real-life experiences of PD.

The aim of this study was to investigate the real-life experiences of PwPD as reflected in online conversations on social media. Through this investigation, we hoped to gain a greater understanding of the important issues affecting PwPD. Additionally, we hoped to elucidate where off-time sits within the wider PD conversation and to raise awareness of the real-world impact of symptoms returning in between medication doses.

## 2. Materials and Methods

### 2.1. Study Design

**Figure 1:**
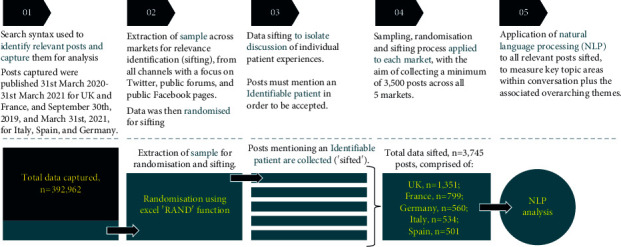
Overview of data sifting process for natural language processing analysis.

This was a qualitative and quantitative netnographic study in which historical PD conversations across public social media were captured and explored to identify key themes and issues for PwPD and their caregivers. A similar methodology to the one described here has been employed previously for different topics [21]. The research was carried out in line with the codes of conduct/guidance laid out for the (pharmaceutical) market research industry, including the British Healthcare Business Intelligence Association (BHBIA) and the European Pharmaceutical Market Research Association (EphMRA). This allows for the undertaking of research using social media data to provide insight; an overview of the process is outlined in Figure 1.

### 2.2. Data Sample/Set

Research keywords covering all potential expressions of the PD patient experience were chosen. Through desk research, we explored social media sources (e.g., Twitter, public forums, and public Facebook pages), as well as other online resources where PD is discussed. The primary aim was to identify suitable key terms relating to the PD landscape, including relevant symptoms, disease terminologies, abbreviations, and treatments, as well as patient-specific language and hashtags. A list of key terms was then constructed, along with a list of additional PD-specific public Facebook pages and forums of interest. The resultant key terms were used to identify relevant posts to capture for analysis. Search syntax was piloted to ensure suitable relevance and refinement, excluding keywords and domains that were a source of noise (Table 1). Search terms were then translated by local language analysts and applied in each country of interest (UK, Germany, France, Italy, and Spain). Systematic data quality assurance was conducted to ensure accuracy at scale. The refined search terms were then applied to historical social media posts with a focus on Twitter, public forums, and public Facebook pages, published between March 31, 2020, and March 31, 2021, for the UK and France, and between September 30, 2019, and March 31, 2021, for Italy, Spain, and Germany. The resulting data sample was then sifted and explored to understand the overall size, shape, and scale of the PD conversation.

### 2.3. Data Sifting

The randomization of posts was achieved by inserting all data into an Excel spreadsheet and using the “RAND” function. Randomized samples of posts from the UK, Germany, France, Italy, and Spain were extracted for manual analysis by expert native-language analysts. Data were then manually sifted to isolate relevant posts about individual patients' experiences for further analysis with a focus on identifying posts from individuals meeting the following criteria: PwPD sharing experiences with having or managing PD; people who are close to PwPD and have intimate knowledge of their experience (close friends, family, or caregiver). The aim was to identify 700 relevant posts from each of the five countries of interest.

### 2.4. Natural Language Processing

Translated coded data were uploaded into natural language processing (NLP) software, and NLP was applied to relevant posts to identify common topics and associated terms within the PD conversation. Further refinement of NLP frameworks was performed to increase accuracy and relevance and develop a code frame. Additional qualitative reading from relevant country- and PD-specific sources that were not included within the initial sifting period was undertaken, to add further detail to concepts identified with NLP and to gauge relative importance and sentiment. These sources were popular PD forums where PwPD discuss the management of their condition, which were not automatically captured using a data capture program due to technical limitations. The content from these PD-specific sources was assessed against the NLP code frame to further validate the code frame and then included as a part of the qualitative analysis (see Section 2.6).

### 2.5. Quantitative Analysis

Results were categorized according to country of interest (UK, Germany, France, Italy, and Spain) and key themes relating to the PD experience were grouped to provide a quantitative view of key term usage on a global level as well as country level.

### 2.6. Qualitative In-Depth Analysis

All results from NLP were consolidated across machine analytics and human-led qualitative analysis to establish key findings. Data from the NLP underwent in-depth qualitative analysis to identify additional nuances and detail within the PD conversation. Analysis on each key topic was performed using top-down and emergent, bottom-up frames as appropriate to identify more nuanced themes within conversation and important context. Manual reading and qualitative analysis were performed, using the same filters and analysis frameworks to ensure consistency.

### 2.7. Contextual Analysis

Additional qualitative reading was performed from relevant PD-specific sources, including public patient support forums, that were not included within the initial scraping period due to technical limitations. This additional qualitative reading was conducted in the same way as the qualitative reading prior to the qualitative in-depth analysis (see Section 2.4). Contextual analysis was conducted in order to elaborate on concepts and add additional detail, particularly where the target sample of 700 posts was not achieved.

## 3. Results

### 3.1. Data Sample

**Figure 2:**
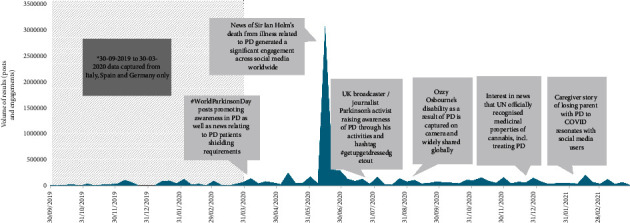
Overview of timing of unique posts and engagements between March 31, 2020, and March 31, 2021, for the UK and France, and between September 30, 2019, and March 31, 2021, for Italy, Spain, and Germany (total number of conversations was 392, 962).

The search terms identified a total of 392,962 posts online between March 31, 2020, and March 31, 2021, for the UK and France, and between September 30, 2019, and March 31, 2021, for Italy, Spain, and Germany. These comprised 392,962 unique posts and 8,101,000 subsequent “engagements” (i.e., responses to unique posts). The unique posts originated in the UK (*n* = 116,576), Germany (*n* = 95,213), France (*n* = 48,306), Italy (*n* = 73,297), and Spain (*n* = 59,570). The UK provided the largest total PD conversation, and the largest peaks in conversation posts occurred in response to the news of Sir Ian Holm's death from illness related to PD and other key events (Figure 2). Most content relating specifically to the PD experience was found on forums (7,500 posts in total), where patients and caregivers discuss disease management and burden, across all stages of the patient journey. Following manual data sifting, a total of 3,745 posts were identified for further analysis (UK, *n* = 1,351; France, *n* = 799; Germany, *n* = 560; Italy, *n* = 534; and Spain, *n* = 501).

### 3.2. NLP Findings

NLP identified several key themes across the PD conversation, comprising time/age, family, disease area, management, and symptoms. The theme of time/age included ways to measure how much time has passed when discussing the patient journey and how old a patient or caregiver is. Family referenced the relationship between patient and caregiver, the family as support network, and the impact of disease on family life and everyday activities. Disease area included conversations and discussions concerning PD awareness, and what it means to have PD, including the day-to-day impact of PD. Management included discussion regarding which treatments or medications were being taken by PwPD, conversations about seeing a specialist or doctor, and discussions regarding side effects and medication adjustment. The theme of symptoms included reference to motor and nonmotor symptoms throughout patient PD journey.

### 3.3. Quantitative Analysis

**Figure 3:**
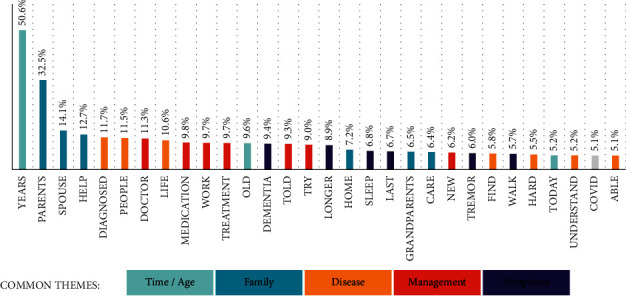
Global topic analysis of common themes: time/age, family, disease area, management, and symptoms.

Key term usage on a global level, as well as country level, was grouped and categorized into key themes relating to the PD experience. In the global topic analysis, the term “years” was used in over 50% of posts in reference to both time and age. Other commonly used terms included “family support,” “disease burden,” and “management” (Figure 3).

**Figure 4:**
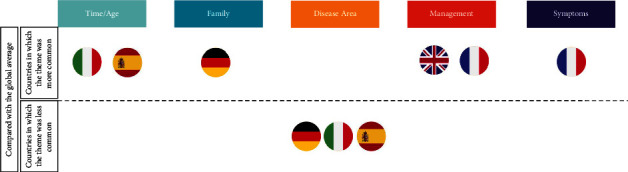
Country differences: how themes in each country compared with the overall global results.

Notable country differences (Figure 4) were found in key discussion points. In the UK, “years” was the most common term, with over 50% of mentions, followed closely by a variety of terms representative of family support. In France, just under 50% of mentions included the term “years” relating to time/age, with management terms such as “treatment” being commonly used throughout posts. The two key themes in Italy that dominated online conversations were time/age and family; the top four topics discussed (“years,” “parents,” “spouse,” and “old”) are all related to the themes of time/age and family. In Spain, “years” was the most common term, with nearly 50% of mentions, followed closely by “parents.” In Germany, two key terms dominated online conversation, “father/mother” and “years,” relating to the family and time/age categories of discussion.

### 3.4. Qualitative In-Depth Analysis

**Figure 5:**
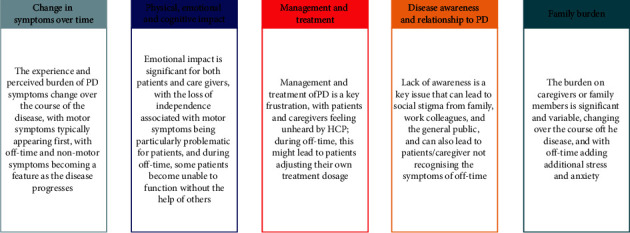
Summary of factors that impair Quality of Life identified from the natural language processing analysis and qualitative deep dive.

During qualitative in-depth analysis of the language PwPD use to describe their experience with PD, more nuanced themes emerged. These are related to how the experience of symptoms changes over time; the frustration of PD management and treatment; the physical, emotional, and cognitive impact of symptoms; and the variability of disease awareness and the relationship to disease, with social stigma identified as an issue; and the caregiver or family burden. Each of these themes was explored in more detail, and a summary of the key points are captured in Figure 5.

#### 3.4.1. Experience of Symptoms over Time

Online conversation reflected that the experienced and perceived burden of PD symptoms changes over the course of the patient journey. Motor symptoms were usually discussed as appearing before nonmotor symptoms—typically tremors, but for some, rigidity. The online conversations indicated that once (usually motor) symptoms become regular and more noticeable, diagnosis is sought, and treatment is typically initiated when symptoms begin impeding daily activities or quality of life. At the same time, some PwPD may experience nonmotor symptoms. However, data collected suggest that PwPD and caregivers found the impact of nonmotor symptoms less pronounced than the impact of motor symptoms at this stage. During the ongoing management of PD, the data suggest that symptoms start to increase, off-time becomes a feature, and nonmotor symptoms increase, compounding physical difficulties. During late-stage management, online conversations provide evidence that the quality of on-time has reduced substantially, the level of care required is amplified, and nonmotor symptoms become difficult to manage, sometimes more than motor symptoms.

This change in the experience of off-time can be reflected in the prioritization of off-time as a key concern. Initially, the burden of off-time on the patient, spouse, and family is discussed online earlier in the patient journey but is not evidenced at the later stages of the journey.

#### 3.4.2. Physical, Emotional, and Cognitive Impact of Symptoms

The data collected indicate that the emotional impact on patients is significant and noted to be primarily connected to an increase in motor symptoms or disability, and the gradual loss of independence. Specifically, as PwPD start to lose their independence due to motor disabilities, they become aware they need help to perform basic tasks, which can lead to depression. On the other hand, the impact of nonmotor symptoms on cognitive processes were seen from the online discussions to be particularly frustrating for caregivers; insomnia and fatigue are commonly discussed and have a physical, emotional, and cognitive impact on the patient and caregiver.

We also observed that there is an impact of off-time, where the experience can be so dramatic that it leaves some patients completely unable to function without the help of others. Nonmotor symptoms can also become much more impactful and compound the difficulties with managing the physical impact of motor symptoms. Furthermore, the online conversations suggest that the COVID-19 pandemic exacerbated the emotional and cognitive impact on PwPD due to social isolation.

#### 3.4.3. Management and Treatment of PD

From the online conversations, we found that management and treatment of PD is a key frustration. PwPD and caregivers were seen to discuss the need for having a good relationship with their HCP and expressed frustration with the lack of adequate management. From these conversations, it was evidenced that patients and caregivers feel that their appointments are rushed and that HCPs do not listen sufficiently to patient needs and concerns. This appeared to be compounded when those who do experience off-time voice their concerns to their neurologist yet feel these are not being taken seriously, which may lead to patients adjusting their own treatment dosage. In addition, the data suggest that during the COVID-19 pandemic, access to supportive treatments, such as physiotherapy, became more difficult, and, as a result, motor symptoms worsened.

#### 3.4.4. Disease Awareness and Relationship to Disease

Social stigma was identified as a key issue in disease awareness. Specifically, we saw that motor symptoms can lead to PwPD experiencing social stigma from both family and the general public. Examples of social stigma included extended family not trusting the PwPD to know their limitations; PwPD having to remind work colleagues about PwPD limitations because of a lack of public awareness; instances of people using motor symptoms of PD to make a joke on social media; and perceived misrepresentation of PwPD in adverts raising awareness or funds for PD.

There also appeared a general lack of awareness of off-time and a lack of concrete terms patients/caregivers used to describe off-time,the implications being that patients may be experiencing off-time but are not recognizing it and delaying seeking treatment until it has a significant impact.

#### 3.4.5. Caregiver or Family Burden

We observed the caregiver or family burden of PD to be significant and variable; over time, as PD progresses and family members grow older, the type of caregiver burden changes. For example, where a PwPD and their partner are in a relationship with an offspring, the partner may take on the role of caregiver when motor symptoms initially manifest. The data show that the burden earlier in the condition often revolves around supporting daily activities that are affected by motor symptoms. Then, as time passes, the grown-up offspring may assume more responsibility, when the partner can no longer cope with the physical requirements.

Consistent with this, the online conversations indicate that the impact of off-time too can be felt not only by the patient but by all those who support them and provide care. Implications can be far reaching, disrupting lives and impacting loved ones, causing adding additional stress and anxiety to the experience.

### 3.5. Contextual Analysis

During contextual analysis, the consolidation of results that particularly focused on treatment and the off-time experience was carried out to establish key findings in this section.

#### 3.5.1. The Priority of Off-Time as a Key Concern Changes over Time

In the period following PD treatment initiation (referenced as approximately 3–10 years following diagnosis), medication provides good or partial control of symptoms, and there is no off-time. Prior to off-time occurring for the first time, it is not typically discussed. Off-time becomes more prevalent in online conversation when motor symptoms start to appear despite medication, but off-time is not always easily identified at this stage. Patients describe periods of treatment “losing effect during the day” or have “lows” or “episodes” but do not often use the term “off-time” when first experiencing these periods. At this stage, PwPD and caregivers still commonly discuss efficacy and side effects. However, as motor symptoms (and some nonmotor symptoms) become pronounced, off-time becomes more noticeable and frequent. The quality and duration of on-time eventually reduces as the overall symptom control worsens until symptoms have advanced so considerably that experience of on-time and off-time are no longer differentiated.

#### 3.5.2. Off-Time Has a Substantial Impact on Both PwPD and Caregivers

Off-time affects not only PwPD but all those who support them and provide care. Some PwPD become “blocked” or “frozen” for periods of the day (30 minutes up to 2 hours referenced). Consequently, they require additional emotional support and assistance with daily tasks, particularly for those that require planning, such as work or social events. The resulting loss of independence forces PwPD to cancel activities and plans, take time off work, or retire early. Nonmotor symptoms that feature in off-periods become burdensome: hallucinations can be particularly distressing; mood swings become more pronounced; depression and anxiety impact quality of life; and sleep disturbances and fatigue impact daily activities, including eating and drinking.

When symptomatic control is poor and nonmotor symptoms become more prominent, the online conversation focuses more on the caregiver burden. Caregivers are essentially “always on call” because PwPD are unable to get through the whole day without support. Sometimes caregivers do not live with the patient and have to “drop everything” if symptoms become upsetting or unmanageable. Nonmotor symptoms, such as mood swings, make caring for the PwPD increasingly difficult, as caregivers may have to “fight” with PwPD to keep them safe or to perform daily activities. Caregivers frequently worry whether a visit to the neurologist or a treatment adjustment is necessary, and the true burden of off-time may be underestimated, particularly for those patients who are not seen frequently by HCPs.

#### 3.5.3. Patients and Caregivers Feel HCPs Do Not Always Listen to Their Concerns regarding Off-Time

Patients and caregivers feel HCPs do not always listen to their concerns regarding off-time, which is compounded by the already poor care that some feel they receive. Concerns around nonmotor symptoms appear the most challenging to communicate, with a treatment change being deemed the only “solution” to their concerns, leaving PwPD and caregivers feeling desperate for more holistic and practical help and guidance.

## 4. Discussion

This investigation of the social media conversations around PD revealed key insights into the impact of PD on those living with the condition and on those who care for them. The issues raised represent those that need to be addressed in order to provide more effective support for PwPD and their caregivers. PwPD and caregivers' conversations online demonstrated that the perception of PD symptoms changes over time, with an increasing emphasis on nonmotor symptoms in line with disease progression. The emotional impact of increasing motor symptoms on patients is significant. The resulting loss of independence impacts not only the patient but also has a physical, emotional, and cognitive impact on the caregiver as well. The caregiver or family burden of PD is significant and variable. The progressive motor symptoms additionally lead to PwPD experiencing social stigma—which emerged as a key theme—resulting from a perceived lack of public awareness of the disease and its limitations. Some PwPD and caregivers express frustration at the quality of care they receive from HCPs over time. A common concern is the desire for more frequent physician consultations. Some PwPD feel that their needs, particularly around quality of life and the management of side effects, should be at the forefront of treatment decisions during these consultations. Patients and caregivers are continually weighing up the burden of side effects alongside treatment efficacy and potential disease progression. Some treatment side effects are, for patients and caregivers, sometimes difficult to differentiate from off-time, resulting in confusion as to when off-time is occurring. As a result, off-time can be challenging for caregivers and some patients to identify, particularly earlier in disease progression or when off-time manifests predominantly as nonmotor symptoms. They need tools to recognize qualitative changes in their experience and to address them.

An important theme that was identified amidst the wider PD discussion was the perception of off-time. Due to the change in prioritization of off-time in online discussion, it could be inferred that patients, caregivers, and HCPs may not be prioritizing managing off-time as the patient progresses along their journey, particularly when patients are dealing with debilitating and worrying side effects of drug treatment. However, off-time increases in priority as PD progresses. As symptoms begin to affect daily activities, caregiver support is required. The impact of off-time can be felt not only by the patient but by all those who support them and provide care. Implications can be far-reaching, disrupting lives and impacting loved ones, adding additional stress and anxiety to the experience.

These study findings indicate a clear scope to improve patient care, with HCPs seeing patients more frequently, spending more time explaining treatment decisions, ensuring side effects are minimal or manageable, and actively managing expectations around potential issues such as off-time. Given the additional burden on healthcare provision, the COVID-19 pandemic has affected many of these goals [22]. However, the widespread use of telemedicine established during the COVID-19 pandemic may provide a future means for PwPD to more readily access health care. Patients and caregivers need to feel that they are on the right treatment and that they are supported as they move through their disease journey.

In addition, scope exists for further education within the wider community around off-time. Despite the knowledge of the PD online community as a whole, caregivers in particular need to be able to better identify off-time in all its forms and the options available to address it. HCPs also need further education to ensure that they too understand the burden of off-time and can recognize and communicate the benefits of available treatment options to improve it. These benefits extend beyond symptom control, with prolonged independence and emotional relief holding equal importance. Caregiver burden should not be forgotten, as the reduction of off-time and the resulting increase in on-time can improve quality of life for all involved in the care of PwPD. It will, however, be important to acknowledge that off-time is one factor in a complex disease and people's disease experience, and, therefore, the impact of off-time can vary significantly. HCPs will need to ensure any communication, support, and resources reflect the multifaceted nature of the off-time experience and related burden.

The online conservation among PwPD provides a valuable insight into issues important to PwPD that can help inform PD care. Information can be gathered from existing social media posts, but social media can equally be employed prospectively as a research tool. For example, Ali et al. used a social media survey to contact patients with Dravet syndrome in order to gather self-reported outcomes of vagus nerve stimulation therapy [23]. Cumulatively, the findings of this and previous studies demonstrate that online platforms such as social media connect and empower individuals with an interest in a particular disease to share information and experiences. Moreover, social media may enable PwPD to express themselves more honestly than when interacting with HCPs in clinical settings, where they may feel more guarded and time is limited. This perhaps, in part, accounts for the disparity between the perceptions of PwPD and those of HCPs of the most important patient care issues. Among PwPD and their caregivers, connecting online appears to be a largely positive experience that allows them to access information and support one another. However, the use of social media may subject users to derogatory language relating to PD and PwPD, reinforce prejudices, and propagate stigma. This emphasizes the need for education among the general public, in order to increase understanding and reduce the stigma that is often still associated with disorders such as PD.

The current study is largely descriptive and limited by its qualitative nature, which is inherently subject to bias. In addition, the methods used to identify text from online posts may not have detected less common themes and may have missed some subtle contexts of the concepts that were captured. For instance, nonmotor symptoms did not appear as a key topic of concern in online discussion despite increasing evidence that nonmotor symptoms do occur in the prodromal phase of the disease [9, 10]. However, the size of the data sample, together with the use of NLP to identify key topic areas and associated themes, mitigates the risk of bias to some extent, while qualitative in-depth analysis allowed the identification of more nuanced themes and important context. Cultural differences between the countries may exist that have not been considered in the interpretation of data. The study is also limited by not including data from other popular social media platforms (e.g., TikTok and Instagram) in the NLP analysis due to technical limitations, and despite comprehensive research and piloting of the search syntax used, it is likely that additional key search terms were missed. It should also be borne in mind that although social media is commonly used, individuals who choose to communicate in this way represent a subgroup of PwPD who have access to social media and who may not necessarily represent the feelings, awareness, and attitudes of the wider PwPD population. In addition, age is an important factor, and, though in our study we take into account the perspectives of caregivers, who are likely younger and do therefore use social media, the majority of PwPD are older and may rely on alternative media, such as TV or newspapers. Therefore, the sample included in our analysis may be skewed towards those with early onset PD. Finally, given the fast-changing and reactive/responsive nature of social media, our data sample would have been influenced by what was going on in the wider world at the time (as illustrated by the observed peaks in conversation over the sample period), and the study's findings should therefore be viewed as a snapshot of the online conversation of PwPD over the defined time period.

## 5. Conclusions

The findings of this study reveal issues of importance to PwPD and caregivers. Briefly, these include the experience and perceived burden of symptoms changing over the course of the disease; a significant emotional impact for both patients and caregivers; patients and caregivers feeling unheard by HCPs; social stigma from family, work, and the general public; and a significant and variable impact on families and caregivers. We also observed these issues to be exacerbated by off-times. Identifying key discussion topics in the PD conversation enables HCPs to devise a more targeted approach to address quality of life concerns in both patients and caregivers. Additionally, the findings may provide a resource to inform the rationale and design of future studies. Future studies' aims might include the elucidation of areas that require targeted education to improve awareness and understanding among the general public and HCPs, and to provide PwPD with the information they need the most; the assessment of the side effects of treatment that most impact the health-related quality of life of PwPD; and investigation into the mental health impacts of living with PD and how these might be addressed.

## Figures and Tables

**Table 1:** Overview of key search terms.

General terms
“Parkinson's disease” or “Parkinsons disease” or #Parkinsons or #Parkinsonsdisease or “Parkinson disease” or #Parkinsondisease
Parkinsonism or parkinsonian or “secondary parkinsonsim”
“Parkin disease”
LRRK2 or ^‡^“leucine-rich repeat kinase 2 gene” or dardarin OR PARK8
“Unified Parkinson's disease rating Scale”
PDQ39 or “Parkinsons disease questionnaire”
6OHDA or “Parkinson's model”
Treatments
Nourianz or nouriast or nouryant or Istradefylline
Kynmobi or “apomorphine hydrochloride”
Carbidopa or Lodosyn
Nuplazid or Primavanserin
AtremoPlus
Levodopa or “L Dopa” or ^‡^“l-dopa” or IPX203
Co-beneldopa or Madopar or “Madopa CR” or “Caramet CR” or Lecado or “Half Sinemet CR” or Sinemet or “Sinemet Plus” or “Sinemet CR” or Duodopa
Co-careldopa or “Apodespan PR”
Duopa or “carbidopa levodopa infusion” or ^‡^“carbidopa-levodopa”
Inbrija or ^‡^“inhaled carbidopa-levodopa” or “inhaled carbidopa levodopa” or “inhaled carbidopa”
Entacapone or Comtan or Comtess or co-careldopa or stalevo or sastravi or stanek
Treatments anchored to: Parkinson's or Parkinsons
“Dopamine agonists” or dopamine or (dopamine NEAR/2 replacement)
Apomorphine or Apokyn or ^‡^“Apo-go” or Dacepton or “Apo-go prefilled pen” or “Dacepton cartridge” or “Apo-go prefilled syringe” or “Dacepton vial”
Pramipexole or mirapex or mirapexin or Pipexus or Glepark or Oprymea or Zentiva
Ropinirole or Requip or Adartrel or “Ralnea XL” or “Requip XL” or “Spiroco XL” or “Ipinnia XL” or “Raponer XL” or “Ropilynz XL”
Rotigotine or Neupro or “Rotigotine transdermal patches”
Amantadine or Gocovri or “glutamate antagonist”
^‡^“MAO-B inhibitors”
Rasagiline or Azilect
Selegiline or Zelapar or “Eldepryl Emsam” or L-deprenyl
Safinamide or Xadago
“COMT inhibitors”
Tolcapone or Tasmar
Opicapone or Ongentys
Anticholinergic or artane
Trihexyphenidyl or Benzhexol or “Benzhexol Trihex”
Ambroxol or mucosolvan or mucobrox or mucol
GyroGlove or GyroGrear or “gyroscopic glove”
LSVT OR “Lee Silverman voice treatment”
Terazosin or hytrin
^‡^“MRI-HIFU” or “magnetic resonance imaging and high-intensity focused ultrasound therapy”
Benzatropin or Cogentin
Proclydine or Kenadrin
Domperidone or motilum
(Treatment or medication) and (“side effects” or limitation ^*∗*^)
Symptoms anchored to: Parkinson's or Parkinsons
Tremor^†^ or shak^†^
Slow^†^ or bradykinesia
Stiff^†^ or rigid^†^
Depress^†^ or anxiety
“Balance problems” or fall or “postural instability”
Anosmia
Insomnia
Memory^†^ or dementia or “Lewy bodies” or “lewy body”
Dyskinesia or spasm or writh^†^ or jerk^†^ or dystonia
Hallucination^†^
Constipat^†^
“Vision problem” or “speech difficulty”
“Swallowing difficulties” or drool^†^ or dribbl^†^
“Restless leg syndrome” or akathisia or akathesia
“Urinary leakage”
Sweat^†^
Dizzy or dizziness
Fatigue or tired^†^ or exhausted
“Off-time” or ^‡^“off-time” or “off period” or ^‡^“off-period” or “off state” or ^‡^“off-state” or “off episodes” or ^‡^“off-episodes” or “wearing off” or “wear off” or ^‡^“wearing-off” or ^‡^“wear-off” “off phase” or ^‡^“off-phase” or (freez^†^ and (gait or movement)) or freez^†^
“On-time” or ^‡^“on-time” or “controlled symptoms” or “honeymoon period” or “control of motor symptoms” or “control of non-motor symptoms” or “control of symptoms” or “controlled symptoms”
Pain or discomfort or uncomfortable
“Low blood pressure” or hypotension
Handwriting or “hand writing”
Hashtags
#Endingparkinsons
#Parkinsonsuk
#PDavengers
#WorldParkinsonsDay
#TeamParkinsons
#ParkinsonsResearch
#Parkinsonsdiseasecure
#Parkinsonsdiseaserecovery
#Parkinsonsdiseasesupport
#Parkinsonsawarenessweek
#Parkinsonsukfindacure
#Parkinsonsukchangingattitudes
#Parkinsonsukfundraiser
#Parkinsonsyoga
#Parkinsonsukcharity
#Parkinsonsexercise
#Parkinsonsawarenessmonth
#Parkinsonsfitness
#Parkinsonssucks
#Parkinsonspower
#Parkinsonswalk
#Parkinsonsdementia
#Hateparkinsonsdisease
#Parkinsonstraining
Exclusion terms
“Police department”
“Democratic party” or “partito democratico”
NYPD

^*∗*^English syntax has been translated by local language analysts and applied in each country. “…” quotation marks = exact phrase; # hash symbol = hashtags which identify posts on a specific topic. ^†^Indicates multiple endings are included e.g. inhibitor, inhibitors. ^‡^Indicates that punctuation must be taken into account.

## Data Availability

Data can be obtained from the corresponding author on reasonable request.
